# Supplementary material for: Genetic Analysis of the Full-Length gag Gene from the Earliest Korean Subclade B of HIV-1: An Outbreak among Korean Hemophiliacs
Source: Viruses. 2019 Jun 11;11(6):545. doi: 10.3390/v11060545 (PMC6631484; doi:10.3390/v11060545)
Supplement: Supplementary file 1 [file viruses-11-00545-s001.pdf]

Position	807	823	940	1012	1151	1264	1281	1674	1707	1722	1897-9	2155	2190	No. PCR amplicon	Date sampled	Year of diagnosis
Consensus	TCA	AAA	TTA	CTT	GAC	ATA	TTC	GAC	GCC	CAG	GTA	ACA	GAA			
Cluster O (n=9)																
DONOR O	..G	...	...	...	...	...	..T	..T	..T	..A	..G	T.G	..G	3	Oct 1991	1990
	..G	...	...	...	...	...	..T	..T	..T	..A	A.G	T.G	..G			1990
	..G	...	...	...	...	...	..T	..T	..T	..A	..G	T.G	..G		Feb 2001	1990
HP-1	..G	...	...	...	...	...	..T	..T	..T	..A	A.G	T..	..G	2	Aug 1991	1991
HP-2	..G	...	...	...	...	...	..T	..T	..T	..A	A.G	T..	..G	3	Jan 1992	1991
HP-3	..G	...	...	...	...	...	..T	..T	..T	..A	A.G	T.G	..G	4	Aug 1992	1991
HP-4	..G	...	...	...	...	...	..T	..T	..T	..A	a.G	T..	..G	3	Oct 1993	1991
	..G	...	...	...	...	...	..T	..T	..T	..A	A.G	T..	..G	2	Oct 2002	1992
HP-6	..G	...	...	...	...	...	..T	..T	..T	..A	..G	T..	..G	3	Sep 1992	1992
HP-8	..G	...	...	...	...	...	..T	..T	..T	..A	A.G	T.G	..G	2	May 1992	1992
HP-10	..G	...	...	...	...	...	..T	..T	..T	..A	A.G	T..	..G	4	Aug 1992	1992
HP-18	...	...	...	...	...	...	...	..T	..T	..A	A.G	T..	..G	2	Apr 1993	1993
	..G	...	...	...	...	...	..T	..T	..T	..A	A.G	T..	..G	2	May 1993	1993
Cluster P (n=13)																
DONOR P	...	G..	C..	A..	.G.	G..	...	...	...	...	...	...	...	5	Oct 1993	1991
HP-5	...	G..	...	...	.G.	G..	...	...	...	...	...	...	...	3	Aug 1993	1992
	...	G..	...	...	...	G..	...	...	...	...	...	...	...		Aug 1993	1992
	...	...	C..	...	.G.	G..	...	...	...	...	...	...	..G	2	Jun 1995	1992
HP-7	...	G..	C..	A..	.G.	G..	...	...	...	...	...	...	..G	4	Jul 1992	1992
HP-9	...	G..	C..	A..	.G.	G..	...	...	...	...	...	T..	...		Dec 2002	1992
HP-11	...	G..	C..	A..	.G.	G..	...	...	...	...	...	G..	...	2	Jul 1992	1992
HP-12	...	G..	C..	A..	.G.	G..	...	...	...	...	...	...	...	2	May 1992	1992
HP-13	...	G..	C..	A..	.G.	G..	...	...	...	...	...	...	...	3	Jul 1992	1992
HP-14	...	G..	C..	A..	.G.	G..	...	...	...	...	.C.	...	...	3	Mar 1992	1992
HP-15	...	G..	C..	A..	.G.	G..	...	...	...	...	...	...	...	3	Mar 2003	1992
	...	G..	...	A..	.G.	...	...	...	...	...	...	...	...		Oct 2013	1992
HP-16	...	G..	C..	A..	.G.	G..	...	...	...	...	...	...	...	2	Dec 1992	1992
HP-17	...	G..	C..	A..	.G.	G..	...	...	...	...	...	...	...	2	Mar 1993	1993
HP-19	...	G..	C..	A..	.G.	...	...	...	...	...	...	...	...		Nov 2002	1993
	...	C..	...	G..	.G.	...	...	...	...	...	...	...	...		Nov 2002	1993
HP-20	...	C..	C..	A..	.G.	G..	...	...	...	...	...	...	...		Oct 2002	1994
Local controls (n=72)																
92HCS10	...	...	...	...	.G.	...	...	...	...	...	...	...	...		Oct 1992	1989
95HSH5	...	...	...	A..	.G.	...	...	...	..t	...	...	...	...	4	May 1995	1989
92LSW5	...	G..	...	...	...	G..	...	...	...	...	...	...	...	2	May 1992	1989
92LSY12	...	G..	...	...	.G.	...	..T	..T	...	...	A.g	...	...		Oct 1992	1989
02LJI8	...	...	...	...	.G.	G..	..T	...	..T	...	...	...	...		Aug 2002	1989
93PJA9	...	...	...	...	.G.	...	...	...	...	...	.C.	...	...	3	Oct 1993	1989
91KJS12	..G	...	...	...	.G.	G..	...	...	...	...	.C.	...	...	5	Dec 1991	1989
94KYJ12	...	...	...	...	.G.	G..	..T	...	..T	...	...	...	...		Dec 1994	1989
93KHS5	...	G..	...	...	...	G..	..T	...	...	...	...	...	...	2	May 1993	1989
93HYH5	...	...	...	...	...	...	...	...	...	...	.C.	...	...	2	May 1993	1989
93BJR3	...	...	...	...	...	...	...	...	...	...	...	...	...		Mar 1993	1989
93AJE5	...	...	...	...	...	...	...	...	...	...	...	...	...		May 1993	1990
93LSK4	...	...	...	...	...	...	...	...	...	...	...	...	...	2	Mar 1993	1990
92KChS3	...	G..	...	A..	.G.	...	...	...	...	...	...	...	...	2	Mar 1992	1990
91YWS12	...	...	C..	A..	.G.	G..	...	...	...	...	...	...	...	2	Dec 1991	1990
92LSH6	..a	G..	...	...	.G.	...	...	...	...	...	...	...	...	5	Jun 1992	1990
95KHB7	...	G..	...	A..	.G.	G..	...	...	...	...	...	...	...		May 1994	1990
92KDG4	...	...	...	...	.G.	...	...	...	...	...	...	T..	...	2	Apr 1992	1990
93JJS10	...	G..	...	...	.G.	G..	...	...	...	...	...	...	...	2	Oct 1993	1990
92KJHg11	...	G..	...	A..	.G.	G..	...	...	...	...	...	...	...		Nov 1992	1990
92HSW8	...	G..	...	...	...	...	...	...	...	...	...	...	...	2	Aug 1992	1990
93YEH1	...	...	...	...	...	...	...	...	...	...	...	...	...	2	Jan 1993	1990
03YGS3	...	...	...	...	...	...	...	...	...	...	...	...	...		Mar 2003	1990
01KJin7	...	...	...	...	...	G..	...	...	...	...	...	T..	...		Jul 2001	1990
910CH8	...	...	...	...	...	G..	...	...	...	...	...	...	...		Aug 1992	1990
92LCS3	...	...	...	A..	.G.	G..	...	...	...	...	...	...	...	2	Mar 1992	1991
92DGi6	...	G..	...	...	.G.	G..	...	...	...	...	...	...	...	2	Jun 1992	1991
92PJB6	...	G..	...	...	A..	...	..T	...	...	...	...	...	...	2	Jun 1992	1991
92JWK3	...	...	...	...	...	...	...	...	...	...	...	T..	...	3	Mar 1992	1991
91HJY7	...	...	...	...	...	G..	...	...	...	...	...	T..	...	2	Jul 1991	1991
99KGS8	...	...	...	...	...	...	...	...	...	...	...	T..	...		Aug 1999	1991
96SNS5	...	...	...	...	...	G..	...	...	...	..A	...	...	..g	2	May 1996	1991
92KYB10	...	...	...	...	...	...	...	...	...	...	...	...	...	2	Oct 1992	1991
92HJiH10	...	...	...	...	...	...	...	...	..t	...	.C.	t..	...		Oct 1992	1991
92KSS12	...	G..	...	A..	.G.	G..	...	...	...	...	...	...	...	2	Dec 1992	1992
93JSH3	..G	...	...	...	...	...	...	...	...	...	...	...	...	2	Mar 1993	1992
92PGJ4	...	...	...	A..	...	G..	...	..T	...	...	...	...	..G		Apr 1992	1992
92JYO12	...	G..	...	...	...	...	...	...	...	...	...	...	...	2	Dec 1992	1992
92CYK6	...	...	...	...	.G.	G..	..T	...	...	...	...	...	...		Jun 1992	1992
92JKS12	...	...	...	...	...	...	...	...	...	...	...	...	...	2	Dec 1992	1992
93KYY2	...	...	...	...	...	...	...	...	...	...	...	...	...	4	Feb 1993	1992
92BSJn9	...	...	...	A..	...	...	.A.	...	...	...	.C.	...	...	2	Jun 1992	1992
92LKH10	...	G..	C..	A..	.G.	...	...	...	...	...	...	...	...	2	Oct 1992	1992
92KJnSo10	...	...	...	...	.G.	G..	...	...	...	...	...	...	...	2	Oct 1992	1992
94KJH10	...	...	...	...	...	...	...	...	...	...	...	...	...	2	Oct 1994	1992
92CNS5	...	...	...	...	...	G..	..T	...	...	...	...	...	...	2	May 1992	1992
93KJGn9	...	...	...	...	...	G..	..T	...	...	...	...	...	...	2	Sep 1993	1993
92KCS6	...	...	...	...	...	...	...	...	...	...	...	...	...	2	Jun 1992	1992
92KJK10	...	...	...	...	...	...	...	...	...	...	...	...	...	2	Oct 1992	1992
93KYS12	...	...	...	...	.G.	...	...	...	...	...	...	...	...	2	Dec 1993	1992
92CIS6	...	...	...	...	...	...	...	...	...	...	...	...	...	2	Jun 1992	1992
93PCky3	...	...	...	...	...	...	...	...	...	...	...	...	...	2	Mar 1993	1992
93KYR2	...	...	...	...	...	G..	..T	...	...	...	...	...	...	6	Feb 1993	1993
93LJSb2	...	...	...	...	.G.	...	...	...	...	...	...	T..	...		Feb 1993	1993
93CHM2	...	...	...	...	...	G..	...	...	...	...	...	...	..G		Feb 1993	1993
94CDH9	..G	...	...	...	...	G..	...	...	...	...	...	...	...	2	Sep 1993	1993
95KJHw4	...	...	...	...	...	...	...	...	...	...	...	...	...	2	Apr 1995	1993
94CYIk8	...	G..	...	...	.G.	G..	...	...	...	...	...	...	...	2	Aug 1994	1993
04KMH5	...	...	...	...	.G.	G..	...	...	...	...	.C.	...	...		May 2004	1993
04LSgH10	...	...	...	...	...	...	..T	...	...	...	.C.	...	...	2	Oct 2004	1994
99HGH5	...	...	...	...	...	...	...	...	..T	...	.C.	...	...	2	May 1999	1994
06YJN7	...	...	...	...	.G.	G..	...	...	..T	...	...	...	...		Jul 2006	1996
01PSJ12	...	...	...	...	...	G..	..T	...	...	...	...	...	...	3	Dec 2001	2001
05JJI1	...	...	...	...	...	...	...	...	..T	...	...	...	..G		Jan 2005	2001
01LHS2	...	G..	...	...	...	...	...	...	..T	...	...	...	...	8	Feb 2001	2001
04KKT7	...	G..	C..	A..	.G.	...	...	...	...	..A	A.g	...	..G	2	Jul 2004	2004
05KBHn5	...	...	...	...	...	...	...	...	..T	...	...	T..	..G		May 2005	2004
05CYgS1	...	...	...	...	...	...	..T	..T	...	..A	...	...	..G		Jan 2005	2004
05KJM2	...	...	...	...	...	...	...	..T	...	..A	...	...	..G		Feb 2005	2004
05KMS2	...	...	...	...	...	...	...	...	...	...	...	...	...		Feb 2005	2004
11JGB7	...	...	...	...	...	...	...	...	..T	...	.C.	...	..G	3	Jul 2011	2011
13PJHg6	...	...	...	...	...	...	..T	...	...	...	...	T..	..G	2	Jun 2013	2013

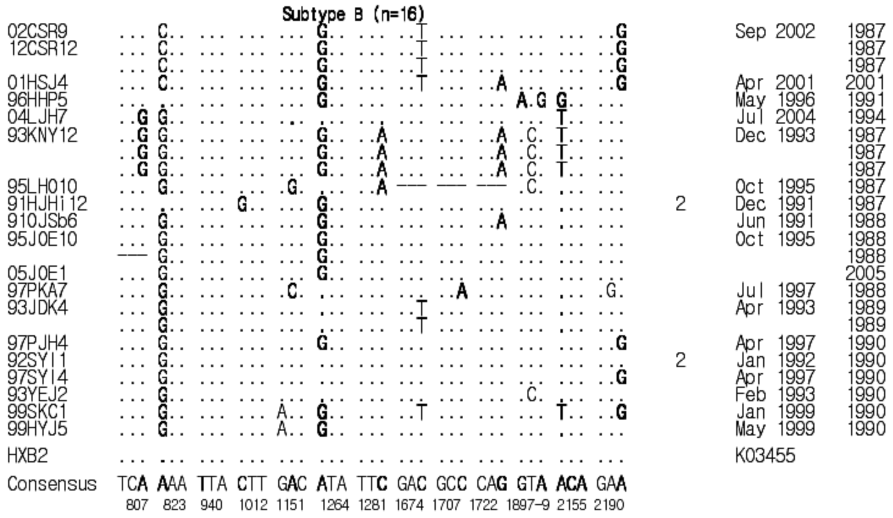

Figure S1: Alignment of 14 signature pattern nucleotides in the 94 patients infected with the Korean subclade of HIV-1 subtype B. Specific nucleotides at positions 9 and 2 were detected in 100% of cases in clusters O and P, respectively, although this high prevalence was not present in the LCs. There was no nucleotide A at position 1897 in three PCR amplicons from HP-6. This difference between clusters O (nine positions) and P (five positions) resulted in a difference in the strength of clustering in clusters O and P. The dot and hyphen indicate the same sequence and deletion compared with consensus sequences, respectively. The nucleotide positions on the uppermost line are based on HIV-1 HXB2 (K03455). Lowercase letters indicated a mixed type with consensus sequences.
